# Carbon Dot-Modified Quercetin Enables Synergistic Enhancement of Charge Transfer and Oxygen Adsorption for Efficient H_2_O_2_ Photoproduction

**DOI:** 10.3390/nano15241856

**Published:** 2025-12-11

**Authors:** Haojie Xu, Zenan Li, Jiaxuan Wang, Fan Liao, Hui Huang, Yang Liu

**Affiliations:** State Key Laboratory of Bioinspired Interfacial Materials Science, Institute of Functional Nano & Soft Materials (FUNSOM), Soochow University, 199 Ren’ai Road, Suzhou 215123, China; xuhaojie11327@163.com (H.X.); 20234014036@stu.suda.edu.cn (Z.L.); 20234014007@stu.suda.edu.cn (J.W.)

**Keywords:** carbon dots, photocatalytic oxygen reduction reaction, hydrogen peroxide, quercetin

## Abstract

Hydrogen peroxide (H_2_O_2_) is a widely used green oxidant, yet its conventional industrial production via the anthraquinone process is energy-intensive and environmentally unfriendly. Photocatalytic oxygen reduction reaction (ORR) presents a sustainable alternative for H_2_O_2_ synthesis, but its practical application is limited by inefficient light absorption, low charge separation efficiency, and sluggish reaction kinetics. In this work, we developed a metal-free carbon-based photocatalyst (QCDs) acquired by modifying quercetin with carbon dots (CDs) for efficient photogeneration of H_2_O_2_. The optimized QCDs achieved a H_2_O_2_ production rate of 1116.32 μmol·h^−1^·g^−1^, which is 40.3% higher than that of pristine quercetin. Comprehensive analysis with transient potential scanning (TPS), transient photovoltage (TPV), and photocurrent transient (TPC) measurements reveal that the photocatalytic ORR follows a two-step single-electron pathway. It is worth noting that CDs not only promote the generation and transfer of photogenerated electrons but also boost oxygen adsorption. Our work demonstrates the synergy of integrating biomass-derived materials with nanostructural engineering and optimizing the system with data-driven approaches for enhanced photocatalysis.

## 1. Introduction

As a versatile and eco-friendly oxidant, hydrogen peroxide (H_2_O_2_) plays an important role in synthetic chemistry, environmental treatment, and biomedical fields [1,2,3,4,5,6,7,8,9,10,11]. However, the dominant industrial method for H_2_O_2_ production—the anthraquinone process—is energy-intensive and relies heavily on toxic organic solvents, resulting in considerable economic and environmental burdens. These limitations have spurred interest in developing sustainable, low-cost, and green synthesis alternatives. Particularly, the photocatalytic oxygen reduction reaction (ORR), which enables H_2_O_2_ generation from solar energy under mild conditions, has attracted increasing attention as a promising strategy [12,13,14,15,16]. Nevertheless, this approach still faces obstacles such as narrow light absorption, inefficient charge separation, and slow surface kinetics. Although noble metal-based catalysts often show high activity, their scarcity and high cost restrict widespread use. Therefore, metal-free carbon-based photocatalysts have been increasingly investigated due to their earth abundance, chemical stability, and environmental compatibility [17,18,19,20,21,22,23,24,25]. Unfortunately, such materials typically exhibit poor charge carrier mobility and limited light-harvesting ability, leading to unsatisfactory photocatalytic ORR efficiency.

Recently, biomass-derived materials have gained popularity as sustainable carbon sources owing to their renewable nature, low cost, and eco-friendly characteristics. Their use not only diminishes dependence on fossil resources but also conforms to green chemistry and circular economy principles. Structural diversity and abundant functional groups in biomass offer a solid basis for designing photocatalysts with tunable properties. Quercetin, a naturally occurring flavonoid found in many plants, features pronounced antioxidant activity, a well-defined molecular structure, and multiple functional groups [26,27,28,29], making it an excellent precursor for constructing high-performance photocatalytic systems. At the same time, carbon dots (CDs) have drawn extensive interest as modifiers in carbon-based catalysis due to their tunable band structure, strong light absorption, and exceptional charge separation promotion capability [30,31,32,33,34]. Incorporating CDs into quercetin-derived carbon frameworks is expected to trigger synergistic effects that markedly improve the efficiency of photocatalytic H_2_O_2_ production.

In this work, a metal-free carbon-based photocatalyst (denoted as QCDs) was fabricated by hybridizing quercetin with CDs for efficient H_2_O_2_ photoproduction. The incorporation of CDs not only broadened light absorption and facilitated charge separation but also helped stabilize key reaction intermediates, thereby substantially boosting H_2_O_2_ yield. Under optimal conditions, the QCD sample delivered a maximum H_2_O_2_ production rate of 1116.32 μmol·g^−1^·h^−1^, exceeding that of the unmodified quercetin-derived catalyst by 40.3%. Through the combination of transient potential scanning (TPS), transient photovoltage (TPV), and transient photocurrent (TPC) techniques, it was confirmed that H_2_O_2_ is generated via a two-step, single-electron ORR route. Importantly, CDs can not only enhance the density of photogenerated charge carriers and the kinetics of interfacial charge transfer but also boosting the oxygen adsorption capacity, all contributing to the enhanced H_2_O_2_ production performance. This study focuses on the key challenges in the field of photocatalytic H_2_O_2_ synthesis. Conventional catalysts often suffer from inefficient separation of photogenerated charge carriers, sluggish surface reaction kinetics, and insufficient active sites. Although carbon-based materials have been extensively investigated, how to precisely design interfacial engineering to simultaneously optimize charge separation and surface reaction processes remains a critical challenge in this field. The innovation of this work lies in constructing a synergistic catalytic system by combining CDs with the natural product quercetin, which achieves dual enhancement in charge separation efficiency and surface reaction activity. The synthesis method is straightforward, and the resulting carbon-based photocatalyst exhibits excellent performance. More importantly, we propose and employ novel characterization techniques, including TPV, TPC, and TPS enabling systematic investigation of catalytic mechanisms that were previously difficult to directly observe. This provides new experimental insights into the structure-performance relationships of such materials.

## 2. Experimental Section

### 2.1. Synthesis of CDs

The detailed synthesis process of CDs is shown in Figure S1 according to the method reported in our previous work [30,31,32,33,34,35,36]. Briefly, 3.0 g of citric acid monohydrate was carbonized in an oil bath at 200 °C for 30 min, followed by the addition of 3.0 g L-cysteine with further carbonization at 150 °C for another 30 min. The resulting sample was named CDs.

### 2.2. Synthesis of QCDs and Quer

The QCD composite photocatalyst was synthesized through a precise thermal processing method. Specifically, 60 mg of CDs and 200 mg of quercetin were accurately weighed and placed into a 100 mL beaker. Then, 50 mL of anhydrous ethanol was added as the dispersion medium to form a homogeneous mixture. The mixture was heated to 80 °C with constant stirring on a thermostatic magnetic stirrer until complete evaporation of the solvent. The resulting solid precursor was thoroughly ground for 30 min in an agate mortar and subsequently transferred into a rectangular ceramic boat. The sample was calcined in a muffle furnace under static air atmosphere, employing a programmed heating rate of 5 °C/min to reach 200 °C, and maintained at this temperature for 2 h. After natural cooling to room temperature, the final product was reground into fine powder with uniform particle size, yielding the QCD composite catalyst.

For the synthesis of Quer, the same experimental procedures were applied except that no CDs was added.

## 3. Results and Discussion

### 3.1. The Characterization and Photoelectric Properties of QCDs and Quer

X-ray diffraction (XRD) analysis was performed to investigate the crystal structure of the materials (Figure S2). Significant differences are observed in the XRD patterns of the QCDs, Quer and Quercetin. Compared to QCDs, several small sharp crystalline diffraction peaks appear in the curves of pristine Quercetin and Quer. Meanwhile, the diffraction peak located at approximately 25° in the trace of QCDs corresponds to the characteristic (100) graphitic plane of CDs [37,38,39,40]. After careful comparison, the diffraction peak positions in QCD matched well with those of Quercetin, indicating that the CDs were well modified onto Quercetin.

**Figure 1:**
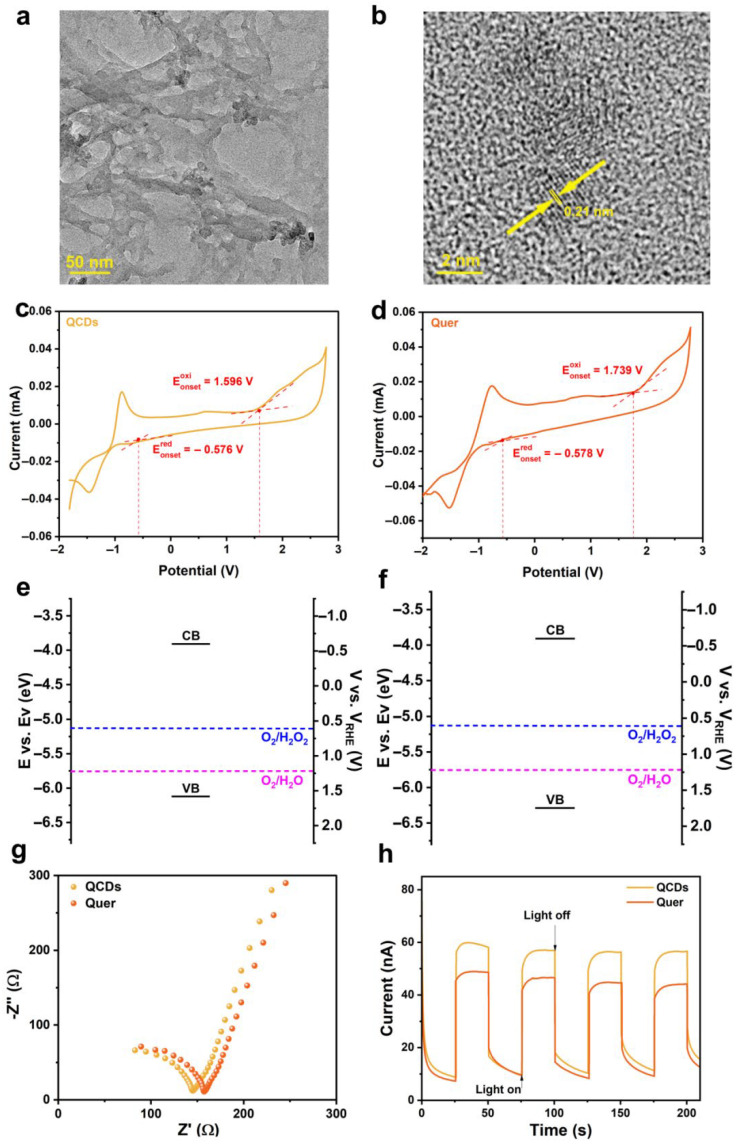
(**a**,**b**) TEM and HRTEM images of QCDs. (**c**,**d**) CV curves in N_2_ saturated TBAP acetonitrile solution; (**e**,**f**) Band structure diagrams obtained from CV; (**g**) EIS plots, and (**h**) TPR curves of QCDs and Quer, respectively.

Figure 1a and Figure S3a,b show the TEM images of QCDs, revealing a flexible sheet-like structure with aggregated dark particles on their surfaces. The high-resolution TEM (HRTEM) image of QCDs (Figure 1b) displays the lattice fringe with a spacing of 0.21 nm in the aggregated dark particles, which matches the (100) plane of graphite, further confirming the synthesis of the QCD composite. In contrast, the morphology of Quer (Figure S3c,d) shows no such aggregated. Figure S3e,f show the TEM images of CDs with different scales, exhibiting the particle size of CDs is around 5–8 nm. Based on the results from XRD and TEM, the carbon-based QCDs and Quer show high similarity in microstructure and morphological features: both exhibit typical graphite-like structural characteristics. It is worth noting that HRTEM observations reveal discrete lattice structures of CDs in QCDs, which are absent in Quer, suggesting that this structural difference may be a key factor affected their catalytic performance.

To further investigate the functional group of the samples, Fourier transform infrared (FTIR) spectroscopy was performed. As shown in Figure S4, the FTIR spectra exhibit several characteristic absorption peaks: O-H (3422 cm^−1^), C-H (2960 cm^−1^), C=O (1750 cm^−1^), C=C (1600 cm^−1^), C-N (1396 cm^−1^), C-S (approximately 1050–1100 cm^−1^), and C-O (1300 cm^−1^). Comparative analysis of the FTIR spectra of QCDs, Quer, and CDs revealed significant differences in the functional group distribution after the loading of CDs. The QCDs exhibited additional functional groups such as C-S and C-N, indicating the introduction of CDs.

X-ray photoelectron spectroscopy (XPS) was employed to investigate the chemical states and electronic structures of the samples. The full-survey XPS spectrum (Figure S5b) confirmed the presence of four elements (C, O, N, and S) in the QCD composite, which are corresponding the characteristic peaks of C 1s and O 1s at 284.6 eV and 532.2 eV, respectively, along with distinct signals in the S 2p (163–164 eV) and N 1s (399–400 eV) regions, indicating the successful incorporation of CDs into the composite system. Figure S5d shows the high-resolution C 1s XPS spectrum of CDs, which displays three characteristic peaks at 284.62 eV (C-C), 286.18 eV (C-O/C-S/C-N), and 288.34 eV (C=O), confirming the existence of carbon skeleton structures. In addition, the high-resolution C 1s spectrum of QCDs (Figure S5e) could be decomposed into three peaks located at 288.24, 286.20 and 284.58 eV, ascribed to the C=O, C−O/C-S/C-N and C-C bonds, respectively. The surface O species analysis was performed using high-resolution XPS O 1s spectroscopy (Figure S5g–i). The high-resolution O 1s spectrum of QCDs (Figure S5h) could be deconvolved into two peaks located 531.5 and 533 eV, attributed to hydroxyl/surface-adsorbed oxygen (OH^−^/O_2_) and reactive oxygen species (O_2_^2−^/O^−^). Quantitative results demonstrate the QCDs possess significantly higher relative content of reactive oxygen species compared to that of Quer [35]. Previous studies have confirmed that such reactive oxygen species could serve as key active sites for the oxygen reduction reaction, effectively promoting oxygen molecule adsorption and activation. Therefore, the elevated concentration of reactive oxygen species in QCDs provides crucial support for enhanced ORR performance. The incorporation of CDs effectively modulates the surface chemical state of QCDs, generating more abundant active oxygen sites that ultimately lead to significantly improved oxygen reduction catalytic performance. The high-resolution N 1s spectrum (Figure S5k) could be deconvolved into two peaks located 401.59 and 400.09 eV, attributed to graphitic N and pyrrolic N, respectively. The high-resolution S 2p spectrum (Figure S5m) could be fitted into two peaks corresponding to 163.31 and 164.58 eV, attributed to S 2p_3/2_ and S 2p_1/2_, respectively. Quantitative results revealed that the binding energies and peak profiles of C and O remained largely unchanged, suggesting their chemical states were well maintained. In contrast, significant alterations were observed in the chemical environments of N and S elements, as evidenced by noticeable shifts in binding energy and changes in peak area ratios. These findings are consistent with the FTIR spectroscopy results, collectively demonstrating that the introduction of CDs markedly altered the functional group distribution and chemical composition of the quercetin-based matrix.

Raman spectra of QCDs and CDs shown in Figure S6 reveal the G band at 1580 cm^−1^ originated from in-plane vibrations of sp^2^-hybridized C, indicating graphitic-like ordered domains, while the D band at 1350 cm^−1^ is associated with carbon lattice defects and disordered structures. Notably, both QCDs (I_D_/I_G_ = 1.03) and CDs (I_D_/I_G_ = 1.01) exhibit prominent D and G bands with intensity ratios (I_D_/I_G_) exceeding 1, suggesting the presence of substantial defects in the CDs. These structural features contribute to the formation of abundant active sites, providing crucial structural foundation for subsequent photocatalytic performance analysis.

To comprehensively understand the energy band structure of the synthesized samples, cyclic voltammetry (CV) measurements for QCDs and Quer were carried out and shown in Figure 1c,d. The CV experiments were conducted in acetonitrile saturated with N_2_, using 100 mM tetrabutylammonium hexafluorophosphate (TBAP) as the supporting electrolyte and ferrocene as an internal standard, ensuring measurement stability and reliability. The band diagrams of QCDs and Quer are shown in Figure 1e and Figure 1f, respectively. After the calculation, the conduction band potential (E_CB_) of QCDs is −0.576 V (reversible hydrogen electrode (RHE)), and its valence band potential (E_VB_) is 1.596 V. While for the energy band structure of Quer, the E_CB_ is −0.578 V and E_VB_ is 1.739 V. The detailed calculation process is displayed in the Supporting Information. These differences in band potentials suggest subtle variations in the electronic configuration and band alignment between the two materials, which may influence their electronic behavior and photocatalytic performance.

Electrochemical impedance spectroscopy (EIS) was further employed to study the internal resistance and charge transfer kinetics of the samples [36]. As shown in Figure 1g, the charge transfer resistance (Rct) of QCDs is 145.3 Ω, while that of Quer is 155.4 Ω, indicating superior electron transfer kinetics in QCDs. Transient photocurrent response (TPR) measurements (Figure 1h) show that QCDs exhibit a 20% higher photocurrent intensity than that of Quer, demonstrating enhanced light-harvesting capability, more efficient separation of photogenerated electron-hole pairs, and reduced recombination rate. These properties contribute to improved charge carrier utilization, thereby enhancing photocatalytic activity.

### 3.2. The Photocatalytic Performance of QCDs and Quer

**Figure 2:**
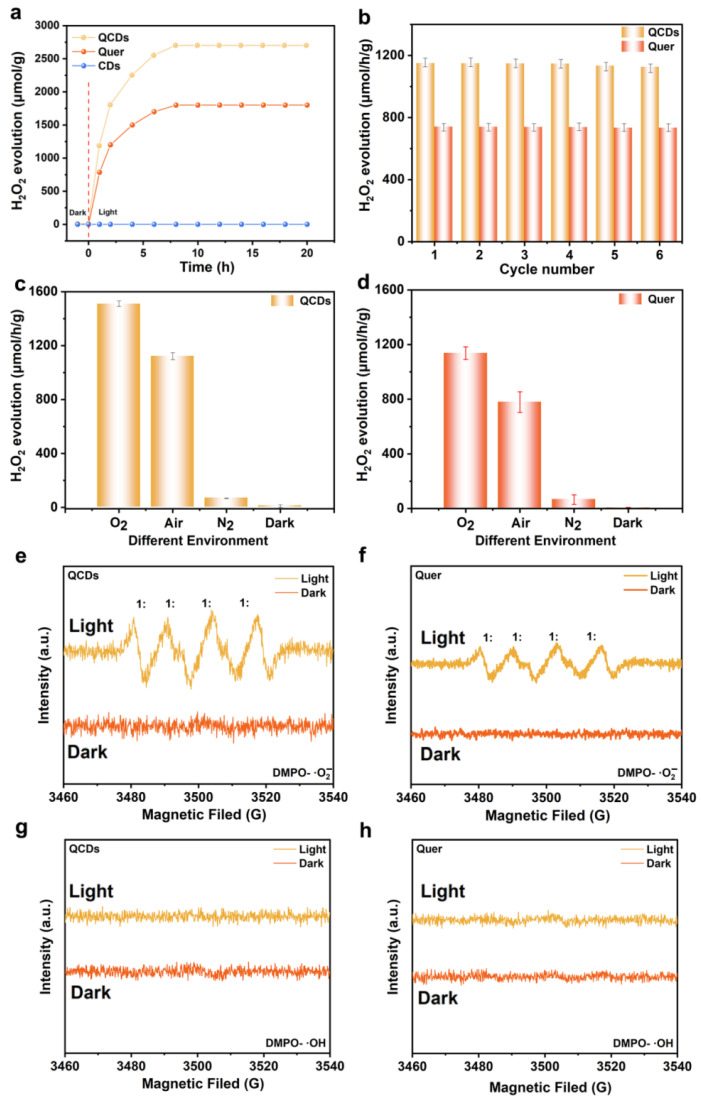
(**a**) The histograms of photocatalytic H_2_O_2_ production under different reaction times catalyzed by QCDs, Quer and CDs. (**b**) Photocatalytic H_2_O_2_ evolution cycles of QCDs. (**c**,**d**) H_2_O_2_ production under different atmospheres for QCDs and Quer, respectively. (**e**,**f**) DMPO spin-trapping EPR spectra for detecting ·O_2_^−^ under dark and light (λ ≥ 420 nm) conditions for QCDs and Quer, respectively. (**g**,**h**) DMPO spin-trapping EPR spectra for detecting ·OH under dark and light (λ ≥ 420 nm) conditions forf QCDs and Quer, respectively.

The photocatalytic performance of obtained catalysts were examined in a closed reactor system using a 300 W Xe lamp as the light source. Photocatalytic production of H_2_O_2_ using photocatalysts under different conditions is shown in Figure S7. To investigate the relationship between the photophysical properties and photocatalytic performance of the materials, we conducted spectroscopic analyses of QCDs and quercetin (Figure S8). The results show that both samples exhibit emission peaks under the same conditions, but the PL intensity of QCDs is significantly lower, indicating more efficient photogenerated charge separation and suppressed non-radiative recombination. In contrast, the stronger PL intensity of quercetin suggests its weaker charge-transfer capability, where more electron-hole pairs recombine radiatively, reducing their availability for catalytic reactions. Furthermore, absorption spectra demonstrate that QCDs possess enhanced light-harvesting capability in the visible region. This observation aligns with the PL results, collectively illustrating that QCDs exhibit superior charge separation efficiency and a broader light absorption range, thereby improving their performance in photocatalytic H_2_O_2_ production. Figure 2a exhibits the H_2_O_2_ evolution ability catalyzed by QCDs, Quer and CDs. As shown, no H_2_O_2_ was detected under dark conditions. During the initial 0–8 h, the reaction was kinetically controlled. After 8 h, the H_2_O_2_ production by QCDs reached equilibrium, and the photocatalytic performance remained stable over time. To evaluate the stability of the catalysts, QCDs and Quer were subjected to five consecutive cycles of photocatalytic H_2_O_2_ production, each lasting 2 h. As shown in Figure 2b, both QCDs and Quer maintained relatively stable activity after the cycling tests. It can be inferred that the introduction of CDs does not significantly affect the ability of the surface functional groups in the quercetin-based material to resist oxidation by photogenerated holes, thereby ensuring the long-term stability of the catalysts.

The photocatalytic performances of QCDs and Quer were systematically investigated under different atmospheres. As shown in Figure 2c,d, under an air environment, QCDs can produce a significant amount of H_2_O_2_ (1116.32 μmol·h^−1^·g^−1^). Figure S9 shows the calibration curve of UV-Vis absorbance at 412 nm vs. different H_2_O_2_ concentrations, as determined by the eFOX reagent colorimetric method. Under the O_2_-saturated atmosphere, the H_2_O_2_ production rate of QCDs further increased to 1500.1 μmol·h^−1^·g^−1^, indicating that oxygen-rich conditions enhance the catalytic efficiency. In contrast, in the N_2_-saturated environment, the H_2_O_2_ production rate decreased significantly to 53.0 μmol·h^−1^·g^−1^, highlighting the crucial influence of oxygen concentration on the photocatalytic reaction. Under the air conditions, the H_2_O_2_ production rate of Quer is 798.2 μmol·h^−1^·g^−1^, which increased to 1165.4 μmol·h^−1^·g^−1^ in an O_2_-saturated atmosphere, markedly higher than that of under air. These results confirm that the photocatalytic H_2_O_2_ production proceeds via the oxygen reduction reaction (ORR) pathway and is strongly influenced by the oxygen concentration.

To further investigate the role of CDs, electron paramagnetic resonance (EPR) spectroscopy using DMPO as a spin-trapping agent was employed to monitor the intermediates generated during H_2_O_2_ production. As shown in Figure 2e,h, no superoxide radicals (·O_2_^−^) or hydroxyl radicals (·OH) were detected for either sample in the dark. Under illumination, however, ·O_2_^−^ was detected, while ·OH remained undetected. These observations suggest that both QCDs and Quer facilitate the two-step single-electron ORR pathway.

### 3.3. Dynamics of Electron Distribution at Catalytic Interfaces Tested by TPS

**Figure 3:**
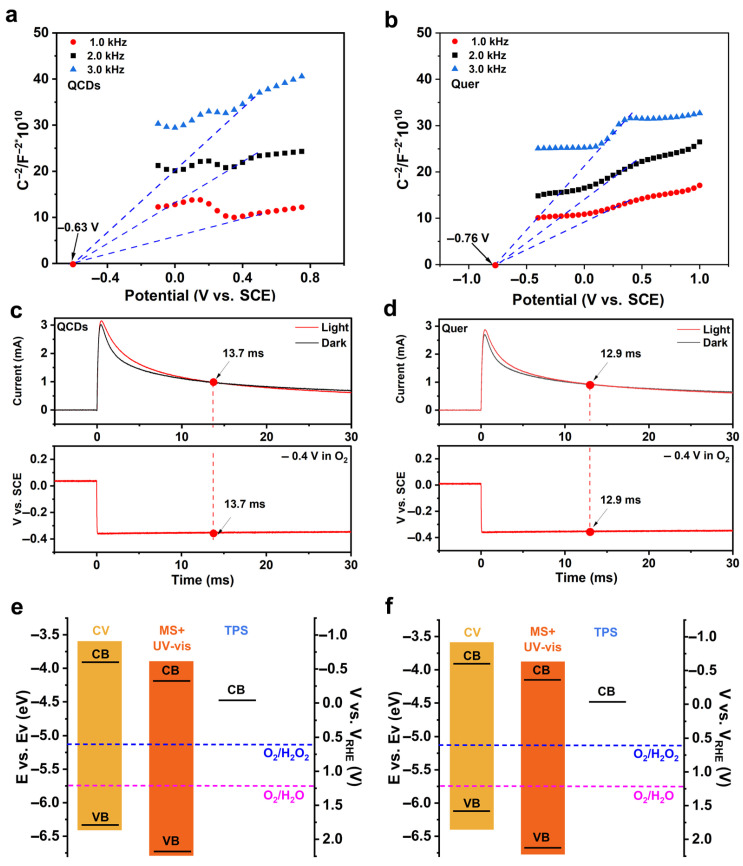
(**a**,**b**) Mott–Schottky plots with different frequencies for QCDs and Quer, respectively. (**c**,**d**) TPS curves under dark and light conditions and corresponding voltage curves of −0.4 V for QCDs and Quer, in O_2_ saturated 0.1 M Na_2_SO_4_ solution. (**e**,**f**) Comparison of bandgap structure of QCDs and Quer in operation condition in O_2_ saturated 0.1 M Na_2_SO_4_ solution, respectively.

To further elucidate the electronic structure of the catalysts, systematic band structure analysis was performed. The optical bandgaps were first determined through UV-Vis diffuse reflectance spectroscopy combined with Tauc plot (Figure S10) analysis. Subsequently, the conduction band potential was precisely measured using Mott–Schottky tests. Based on semiconductor band theory, the corresponding valence band potential was derived through calculation, ultimately establishing a complete band structure model of the catalysts. These results provide crucial theoretical foundation for understanding the photocatalytic reaction mechanism of the materials. Through analysis of the Mott–Schottky plots, the flat-band potential (E_fb_) of QCDs was estimated to be −0.63 V vs. SCE (Figure 3a), while the conduction band minimum (E_CB_) of Quer was determined to be −0.76 V vs. SCE (Figure 3b). Furthermore, considering that the E_CB_ of an n-type semiconductor is typically 0.1 V to 0.3 V more negative than its E_fb_, the E_CB_ values of QCDs and Quer were calculated to be −0.17 V and −0.3 V vs. RHE, respectively, using the relation E_CB_ = E_fb_ − 0.2 V. These results indicate that the incorporation of CDs did not significantly alter the energy level structure. The energy level diagrams of QCDs and Quer are presented in Figure 3e,f, revealing that the band structures of both materials satisfy the potential requirements for ORR.

Transient photovoltage spectroscopy (TPS) was utilized to probe the interfacial charge distribution dynamics during photocatalytic processes [37]. The experimental setup is illustrated in Figure S11. Measurements were performed in a 0.1 M Na_2_SO_4_ electrolyte saturated with N_2_ or O_2_ to maintain well-defined reaction environments. Initial cyclic voltammetry (CV) scans (Figure S12) were conducted to identify a suitable working potential. A potential of −0.4 V was chosen for subsequent TPS analysis, as it lies outside the range of Faradaic processes, thereby minimizing contributions from side reactions. TPS profiles were then acquired under both dark and illuminated conditions to evaluate light-induced changes in interfacial charge behavior.

In semiconductor-electrolyte systems, the application of bias potentials induces electron or hole injection, shifting the interfacial Fermi level and modifying band bending. The flat-band potential, a key parameter for understanding charge transfer, corresponds to the applied potential where band bending is eliminated and the space charge region is minimized. As illustrated in Figure 3c, the intersection of photocurrent curves under light and dark conditions defines the operational flat-band potential. At this point, the photovoltage signal in the V–t curve reflects the actual band alignment under catalytic conditions, offering a more realistic picture than conventional ex situ measurements.

Using this approach, the band structures of QCDs and Quer were determined in an O_2_-saturated electrolyte. Figure 3c,d present the current crossover points and corresponding voltage transients, while Figure 3e,f compare the derived band alignments. Under operational conditions, the conduction band (CB) positions were measured at 0.037 V and 0.084 V (vs. RHE) for QCDs and Quer, respectively. Although these CB levels are thermodynamically favorable for proton reduction, their practical utility is limited by the operational potential window.

**Figure 4:**
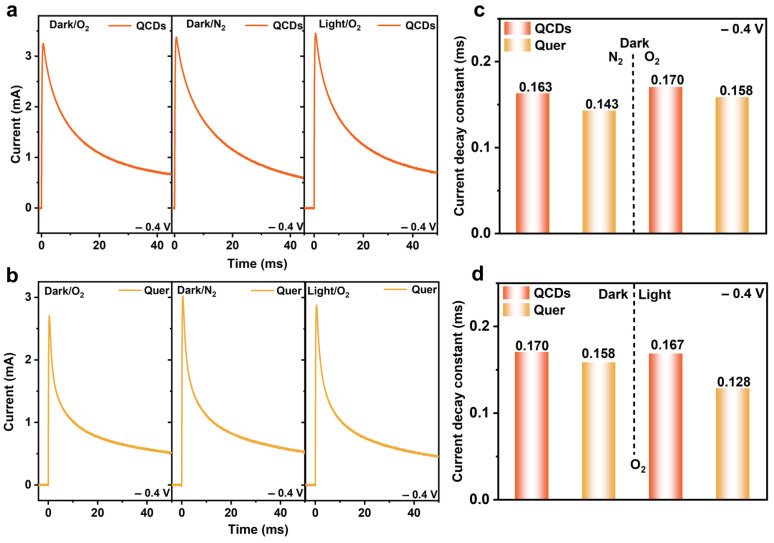
(**a**) TPS curves with −0.4 V applied on QCDs in dark/light under N_2_/O_2_. (**b**) TPS curves with −0.4 V applied on Quer in dark/light under N_2_/O_2_. (**c**) Comparison of current decay constant obtained by TPS curves in dark under N_2_/O_2_ for QCDs and Quer. (**d**) Comparison of current decay constant obtained by TPS curves in dark/light under O_2_ for QCDs and Quer.

At an applied bias of −0.4 V, electrons constitute the majority carriers, and the oxygen reduction reaction (ORR) becomes dominant. The current decay constant (τ), extracted from the transient responses in Figure 4a, is governed by the RC time constant (τ = R × C) and reflects the competition between O_2_ adsorption and its electrochemical reduction. Specifically, O_2_ adsorption increases the interfacial capacitance, leading to a larger τ, whereas the consumption of adsorbed oxygen via ORR and the formation of conductive intermediates facilitate charge transfer, thereby reducing τ. Under dark conditions in an O_2_-saturated electrolyte, τ increased relative to the N_2_-saturated case—from 0.163 to 0.170 ms for QCDs and from 0.143 to 0.158 ms for Quer—indicating enhanced interfacial capacitance due to O_2_ adsorption. Moreover, QCDs exhibited a higher τ value than that of Quer, implying a greater charge-storage capacity associated with oxygen ad species.

The τ values extracted from TPS measurements under O_2_ (Figure 4d) further reveal different responses to light irradiation. For QCDs, τ decreases only slightly from 0.170 to 0.167 ms upon illumination, implying that although surface-adsorbed O_2_ is activated, a high level of O_2_ coverage and capacitance is largely preserved. In contrast, the τ of Quer drops more markedly from 0.158 to 0.128 ms under light, indicating that photogenerated electrons mainly accelerate the consumption of adsorbed O_2_ and result in a lower steady-state capacitance. Combined with the photocatalytic activity results, these observations suggest that the ability of QCDs to maintain a high density of adsorbed and moderately activated O_2_ molecules is beneficial for efficient two-electron ORR to H_2_O_2_, and may be one of the key factors underlying their higher catalytic performance compared with Quer [38].

Based on the experimental evidence, both QCDs and Quer demonstrate pronounced oxygen adsorption capacity. The incorporation of CDs further promotes the activation of adsorbed oxygen species, leading to accelerated charge transfer and enhanced reaction kinetics under light irradiation. The variation in adsorption and activation performance between the two materials may be attributed to differences in surface states, ligand configurations, and the injection pathways of photogenerated electrons. Following CD modification, QCDs exhibit increased electron density, superior charge transport behavior, and a higher interfacial ORR rate, as confirmed by transient charge dynamics analysis.

### 3.4. Electron Transfer Pathways Analyzed Using TPC

**Figure 5:**
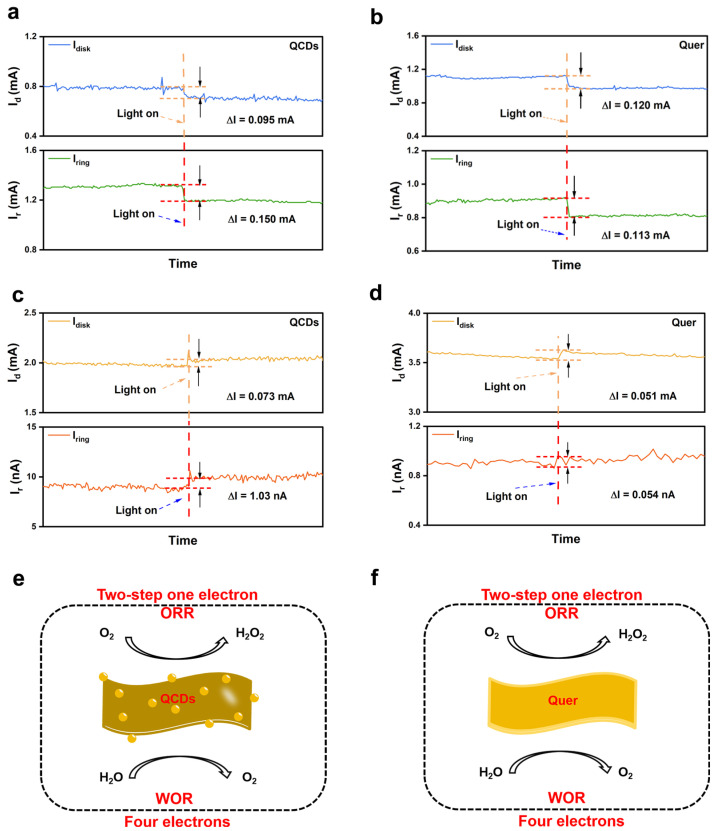
I-t curves of QCDs (**a**) and Quer (**b**) under dark and light (λ ≥ 420 nm) conditions in O_2_-saturated 0.1 M TBAP acetonitrile solution (40 mL) containing 2 mg/L H_2_O. I-t curves of QCDs (**c**) and Quer (**d**) under dark and light (λ ≥ 420 nm) conditions in N_2_-saturated 0.1 M Na_2_SO_4_ solution (40 mL). (**e**,**f**) The reaction mechanism diagram of QCDs and Quer, respectively.

The electron transfer pathway during H_2_O_2_ formation was examined using rotating ring-disk electrode (RRDE) chronoamperometry under varied environments (Figure S13) [39]. In acetonitrile with 2 mg/L H_2_O, the oxygen reduction reaction (ORR) by QCDs proceeded with an electron transfer number of 1.03 (Figure 5a) which is consistent with a single-electron route. Similarly, Quer exhibited a value of 1.17 (Figure 5b), also approximating a one-electron process, corroborating the dominance of the 1e^−^ ORR mechanism in both catalysts.

To assess the hole-driven half-reaction, the electron transfer number for the water oxidation reaction (WOR) was evaluated in N_2_-saturated 0.1 M Na_2_SO_4_. While the blank electrode displayed minimal photoresponse at the disk, the Pt ring electrode maintained a stable photocurrent signal. Under illumination, QCDs and Quer exhibited electron transfer numbers of 4.13 and 4.17, respectively (Figure 5c,d), which are both indicative of a four-electron water oxidation pathway.

Collectively, these RRDE results confirm that CD incorporation preserves the intrinsic ORR mechanism, maintaining the single-electron reduction of oxygen without shifting the reaction pathway. Meanwhile, the hole-mediated water oxidation consistently follows a four-electron transfer process across both materials, indicating that CDs enhance catalytic activity without altering the fundamental reaction routes.

### 3.5. The Interfacial Charge Transfer Kinetics of Samples Tested by TPV

**Figure 6:**
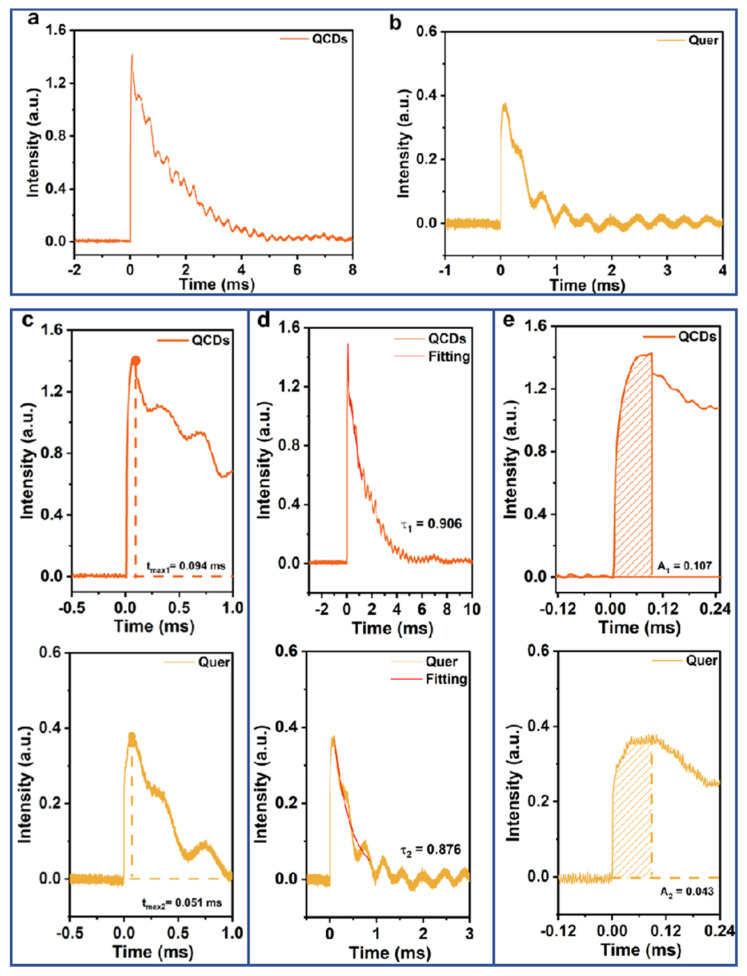
TPV curves of QCDs (**a**) and Quer (**b**). (**c**) T_max_, (**d**) τ, and (**e**) A of QCDs and Quer.

Transient photovoltage (TPV) measurements were performed to probe the interfacial charge transfer kinetics of the catalysts (Figure S14). Critical kinetic parameters—including the photogenerated carrier extraction time (t_max_), charge decay constant (τ), and peak extraction capacity (A)—were derived from the TPV profiles (Figure 6a,b) to evaluate charge separation and recombination behavior [40,41,42,43].

Analysis of the amplified and integrated TPV signals revealed a longer extraction time for QCDs (t_max1_ = 0.094 ms) compared to Quer (t_max2_ = 0.051 ms) (Figure 6c), suggesting a moderately slower initial charge separation under illumination in the CD-modified sample. However, QCDs exhibited a larger decay constant (τ_1_ = 0.906 ms) relative to Quer (τ_2_ = 0.876 ms) (Figure 6d), indicating suppressed charge recombination. Moreover, the peak extraction capacity of QCDs (A_1_ = 0.107) exceeded that of Quer (A_2_ = 0.043) (Figure 6e), reflecting a greater accumulation of photogenerated charges in QCDs.

The effective electron number (n_e_ = A × τ/t_max_) was calculated to further compare the photocatalytic capability. The higher n_e_ value of QCDs (1.031) over Quer (0.738) underscores the enhanced interfacial charge utilization in the former. This improvement is attributed to the more efficient consumption of photogenerated holes via functional groups introduced by CDs, which mitigates recombination and promotes charge availability for surface reactions. These TPV results consistently explain the superior photocatalytic performance of QCDs during H_2_O_2_ production.

**Figure 7:**
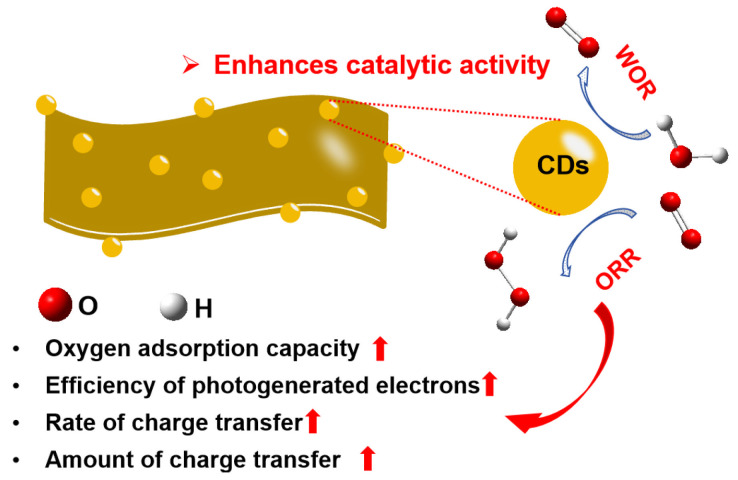
Schematic diagram of photocatalytic ORR with QCDs for the H_2_O_2_ production and the influence of CDs in the photocatalytic process.

A comparative evaluation of the photocatalytic performance of QCDs and Quer demonstrates that the incorporation of CDs markedly enhances the photocatalytic efficiency of QCDs. TPS, TPV, and TPC measurements consistently reveal that CDs endow QCDs with more efficient electron–hole separation, stronger oxygen adsorption, and facilitated O_2_ activation, collectively contributing to accelerated charge-transfer kinetics and improved ORR activity. Under illumination, QCDs display a higher photocurrent response and superior charge-transport behavior compared to Quer, confirming the pivotal role of CDs in boosting overall catalytic performance. As illustrated schematically in Figure 7, CDs not only enhance light absorption and electron transport within the quercetin-based framework, but also improve system stability and interfacial reaction dynamics. These synergistic effects highlight the potential of integrating biomass-derived molecules with carbon nanostructures to develop high-performance photocatalysts, providing a promising direction for future catalyst design.

## 4. Conclusions

In this study, we developed a metal-free carbon-based photocatalyst (QCDs) for efficient H_2_O_2_ production by modifying the CDs on quercetin [44,45,46,47]. The optimized QCDs achieved a H_2_O_2_ production rate upped to 1116.32 μmol·h^−1^·g^−1^, which is 40.3% higher than that of pristine quercetin. As shown in Table S1, the QCDs catalyst in this study demonstrates significantly superior performance compared to others' carbon‑based photocatalysts reported in the literatures [48,49,8,50,51]. The TPV, TPC and TPS measurements were used to confirm the photocatalytic ORR, which followed a two-step single-electron pathway. Systematic investigation of the interfacial charge distribution dynamics, oxygen adsorption and activation mechanisms, and charge transfer pathways throughout the reaction process demonstrated that the incorporation of CDs significantly enhances the catalytic activity of the system. Our work demonstrates the synergy of integrating biomass-derived materials with nanostructural engineering and optimizing the system with data-driven approaches for enhanced photocatalysis.

## Data Availability

All the data generated or analyzed in this manuscript are available in the article.

## Supplementary Materials

The following supporting information can be downloaded at: https://www.mdpi.com/article/10.3390/nano15241856/s1, Figure S1: The synthesis route of CDs and QCDs. Figure S2: XRD patterns of Quercetin, QCDs and Quer. Figure S3: TEM images of QCDs (a,b), Quer (c,d) and CDs (e,f) with different scales. The comparative morphology of the sample before (g) and after (h) the reaction. Figure S4: FTIR spectra of QCDs, CDs and Quer. Figure S5: Full survey XPS spectra of CDs (a), QCDs (b) and Quer (c). High-resolution C 1s XPS spectra of CDs (d), QCDs (e) and Quer (f). High-resolution O 1s XPS spectra of CDs (g), QCDs (h) and Quer (i). High-resolution N 1s XPS spectra of CDs (j) and QCDs (k). High-resolution S 2p XPS spectra of CDs (l) and QCDs (m) Figure S6: (a) Raman spectrum of QCDs, (b) Raman spectrum of Quer. Figure S7: Photocatalytic generation of H_2_O_2_ using photocatalysts under different conditions. (a) Hydrothermal temperature, (b) reaction time, and (c) CDs dosages. Figure S8: (a,b) PL emission spectra of QCDs and Quer. (c,d) UV-Vis absorption spectra of QCDs and Quer. Figure S9: The fitted curve of UV-Vis absorbance at 412 nm vs. different H_2_O_2_ concentrations by the titanium sulfate method. Figure S10: (a,b) UV-Vis absorption spectra of QCDs and Quer. Tauc plots for (c) QCDs and (d) Quer. Figure S11: Schematic diagram of TPS. Figure S12: The CV curves of QCDs (a) and Quer (b) in 0.1 M Na_2_SO_4_ solution. Figure S13: Schematic diagram of TPC. Figure S14: Schematic diagram of TPV. Table S1. Performance comparison of carbon based photocatalysts.
