# Supplementary material for: Carbon Dot-Modified Quercetin Enables Synergistic Enhancement of Charge Transfer and Oxygen Adsorption for Efficient H2O2 Photoproduction
Source: Nanomaterials (Basel). 2025 Dec 11;15(24):1856. doi: 10.3390/nano15241856 (PMC12735901; doi:10.3390/nano15241856)
Supplement: Supplementary file 1 [file nanomaterials-15-01856-s001.zip › nanomaterials-4017987-supplementary.pdf]

Supporting information

Carbon dot-modified quercetin enables synergistic enhancement of charge transfer and oxygen adsorption for efficient H_2O_2 photoproduction

Haojie Xu, Zenan Li, Jiaxuan Wang, Fan Liao*, Hui Huang*, Yang Liu*

State Key Laboratory of Bioinspired Interfacial Materials Science, Institute of Functional Nano & Soft Materials (FUNSOM), Soochow University, 199 Ren'ai Road, Suzhou, 215123, Jiangsu, China.

E-mail addresses: fliao@suda.edu.cn (Fan Liao); hhuang0618@suda.edu.cn (Hui Huang); yangl@suda.edu.cn (Yang Liu)

1. Additional methods

1.1 Material characterization

The microstructures of materials morphology were determined by a FEI Talos 200x transmission electron microscope (TEM) under an acceleration voltage of 200 kV. The powder X-ray diffraction (XRD) pattern was carried out by PIXcel4b X-ray diffractometer (Empyrean, Holland Panalytical) with Cu Kα radiation (0.154073 nm). Hyperion spectrometer (Bruker, Germany) was employed to determine Fourier transform infrared (FTIR) spectrum over a scan range of 400-4000 cm^{-1}. X-ray photoelectron spectroscopy (XPS) was measured with Thermo Scientific K-Alpha photoelectron spectroscope with a monochromatic Al Kα X-ray source as the excitation source ($h\upsilon$ = 1486.6 eV). A C 1s peak at 284.6 eV of adventitious carbon was employed for calibration. The UV-Vis adsorption spectrum at room temperature was acquired using a UV/visible/NIR spectrophotometer (lambda 750, Perkinelmer) with a wavelength range of 250 - 800 nm. Electron spin-resonance spectroscopy (ESR) measurements were performed on Bruker EMXplus-6/1 to analyzed H_2O_2 evolution process. Electrochemical measurements were conducted on the CHI 760C workstation (CH Instrument, Shanghai, China).

1.2 Electrochemical impedance spectroscopy (EIS) measurement

EIS measurements were made in an open=circuit potential (OCP) with a frequency range of 1000 Hz to 3000 Hz. A xenon lamp with 100 W was used as the light source, and the light response time curve was obtained in 0.1 M sodium sulfate (Na_2SO_4 0.1 M, pH = 6.8). The working electrode was a glassy carbon electrode (GCE) coated with catalysts. The counter electrode and reference electrode were a carbon rod and a saturated calomel electrode (SCE).

1.3 Transient photocurrent response (TPR) measurement

TPR measurements were performed using a three-electrode system. The test was carried out at open-circuit potential with a 30-second optical on/off cycle, provided by a 100 W Xenon lamp cold light source ($\lambda \geq 420$ nm). GCE was used as the working electrode. A carbon rod was used as the counter electrode, and SCE was used as the reference electrode.

1.4 HOMO/LUMO calculation

The HOMO and LUMO were determined by a cyclic voltammogram (CV) curve. The CV curve was obtained in N_2 saturated acetonitrile solution with 0.1 M Tetrabutylammonium hexafluorophosphate (TBAP), in which 5 mg ferrocene was used as the external standard. The working electrode is the GCE with sample. The counter electrode is carbon electrode and the reference electrode is Ag/Ag^+ electrode (AgCl dissolved in acetonitrile). The scan rate is 10 $mV \cdot s^{-1}$. Therefore, the HOMO and LUMO can be obtained by following equations:

$$E_{HUMO} = -(E_{onset}^{ox} - E_{ferrocene} + 4.8) \tag{S1}$$

$$E_{LUMO} = -(E_{onset}^{red} - E_{ferrocene} + 4.8) \tag{S2}$$

LUMO selects the onset potential of the last oxidation peak, and HOMO selects the onset potential of the first reduction peak. The Ag/Ag^+ electrode potential was then converted to a relatively reversible hydrogen electrode potential.

1.5 Transient photovoltage (TPV) measurement

The TPV system uses a pulsed laser with a wavelength of 355 nm and a pulse width of 5 ns. The signal of TPV was detected through the change in output voltage. One of the prerequisites for this is that the current value in the external circuit must be close to zero (<< 1 nA). Only in this case, the change in output voltage can reflect the change in the amount of accumulated charge of the photoelectrode, and thus reflect the property parameters of the photoelectrode and the charge transfer dynamics of the interface. Therefore, through the in situ TPV test, we can analyze the process of photo-generated charge extraction and recombination within a wide time window.

1.6 Transient potential scanning (TPS) measurements

TPS measurements were conducted on a home-made measurement system. The circuit contains a 470 μF capacitor with an adjustable voltage to charge the capacitor. GCE was used as working electrode, Pt wire was regarded as counter electrode, and SCE was used as reference electrode, in N_2/O_2 saturated 0.1 M Na_2SO_4 solution as electrolyte. 6.3 μL of solution (1 mL catalyst after freeze-drying with 1 mL 0.5 % nafion solution) was dropped onto the electrode surface. The current and voltage curves in the circuit were recorded. The voltage and current values were measured with data acquisition rate of 100,000 per second. The depletion layer varies continuously when a negative potential is applied to an n-type semiconductor, and a positive potential to a p-type semiconductor, until a flat band state is reached.

1.7 Determination of electron transfer number

The calculation formula is shown as follow:

$$n = \frac{4\Delta I_d}{\Delta I_d + \frac{\Delta I_r}{N}} \tag{S3}$$

ΔI_d and ΔI_r correspond to the current difference on the disk and ring electrodes in light, respectively, and N equals to 0.43 representing the collection coefficient of the RRDE electrode. The rotating ring disk electrode covered with the sample on the disk was used as the working electrode, and the saturated calomel electrode and the Pt wire were used as the reference and counter electrodes, respectively. The scan rate is 10 $mV{\cdot}s^{-1}$.

1.8 Measurement of photocatalytic properties

5 mg catalyst was added into 40 mL optical-grade quartz bottle and mixed with 10 mL ultrapure water. The thickness of the quartz bottle is 1 mm to ensure that the light transmittance is greater than 95 %. Then it was covered with rubber stopper and plastic film for seal. In addition, the suspension was treated wtih ultrasound for 30 min. The suspension was continuously stirred under visible light ($\lambda \geq 420$ nm) with the average intensity 116.3 $mW{\cdot}cm^{-2}$ by a multi-channel photochemical reaction system (CEL-LAB200E7).

The test for photocatalytic performance under saturated O_2 or saturated N_2 was carried out in a continuously blow high-purity O_2 or high-purity N_2 into the solution for 30 min. After that, the test was started.

The cycling experiments were conducted in ultrapure water (10 mL) under visible-light

irradiation ($\lambda \geq 420$ nm) for 4 h under ambient atmospheric conditions. After each cycle, the catalyst was recovered by centrifugation, washed repeatedly with ultrapure water, and dried at 60 °C. The dried catalyst was then weighed to ensure mass consistency before being reused in the subsequent photocatalytic reaction. This procedure was repeated for a total of six cycles to assess the catalyst's stability and reusability.

2. Additional Figure

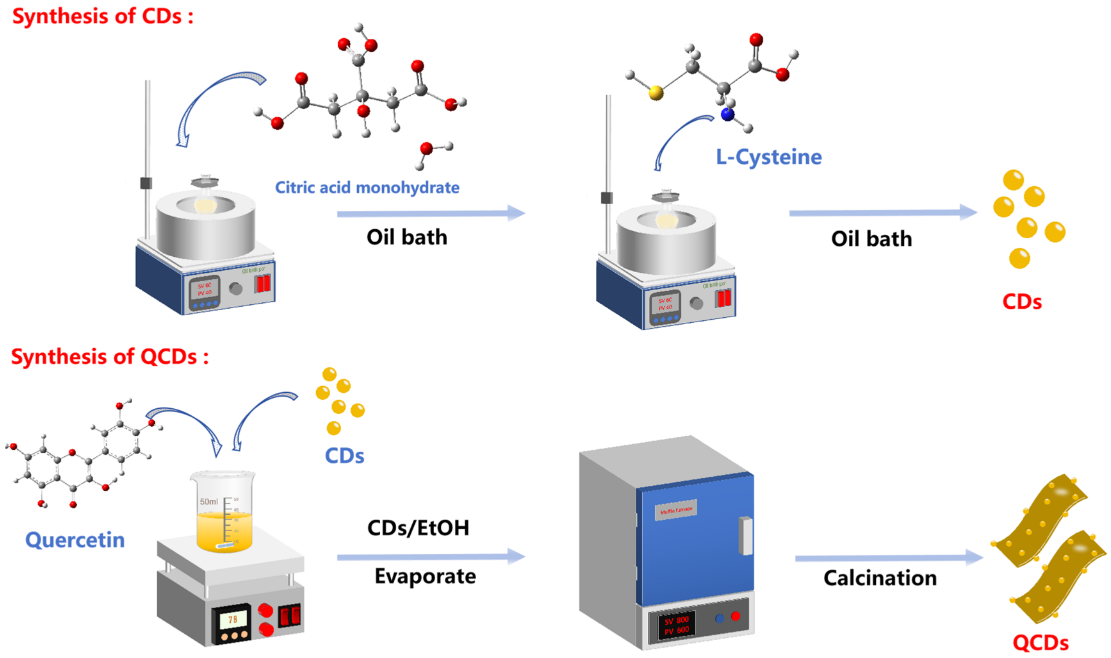

Figure S1 The synthesis route of CDs and QCDs.

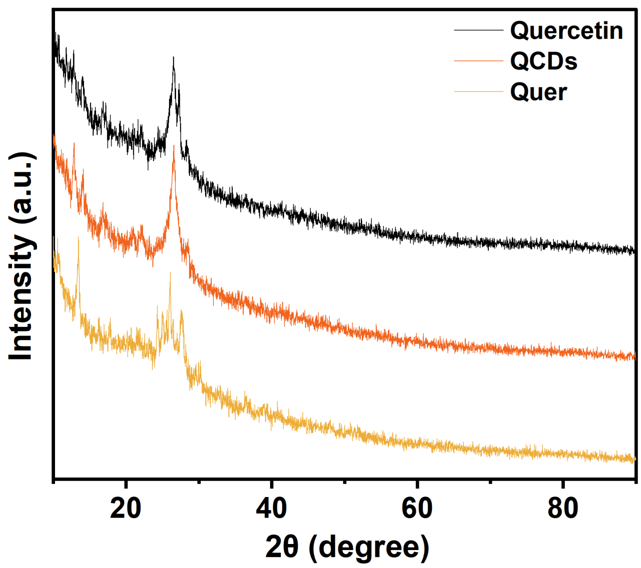

Figure S2 XRD patterns of Quercetin, QCDs and Quer.

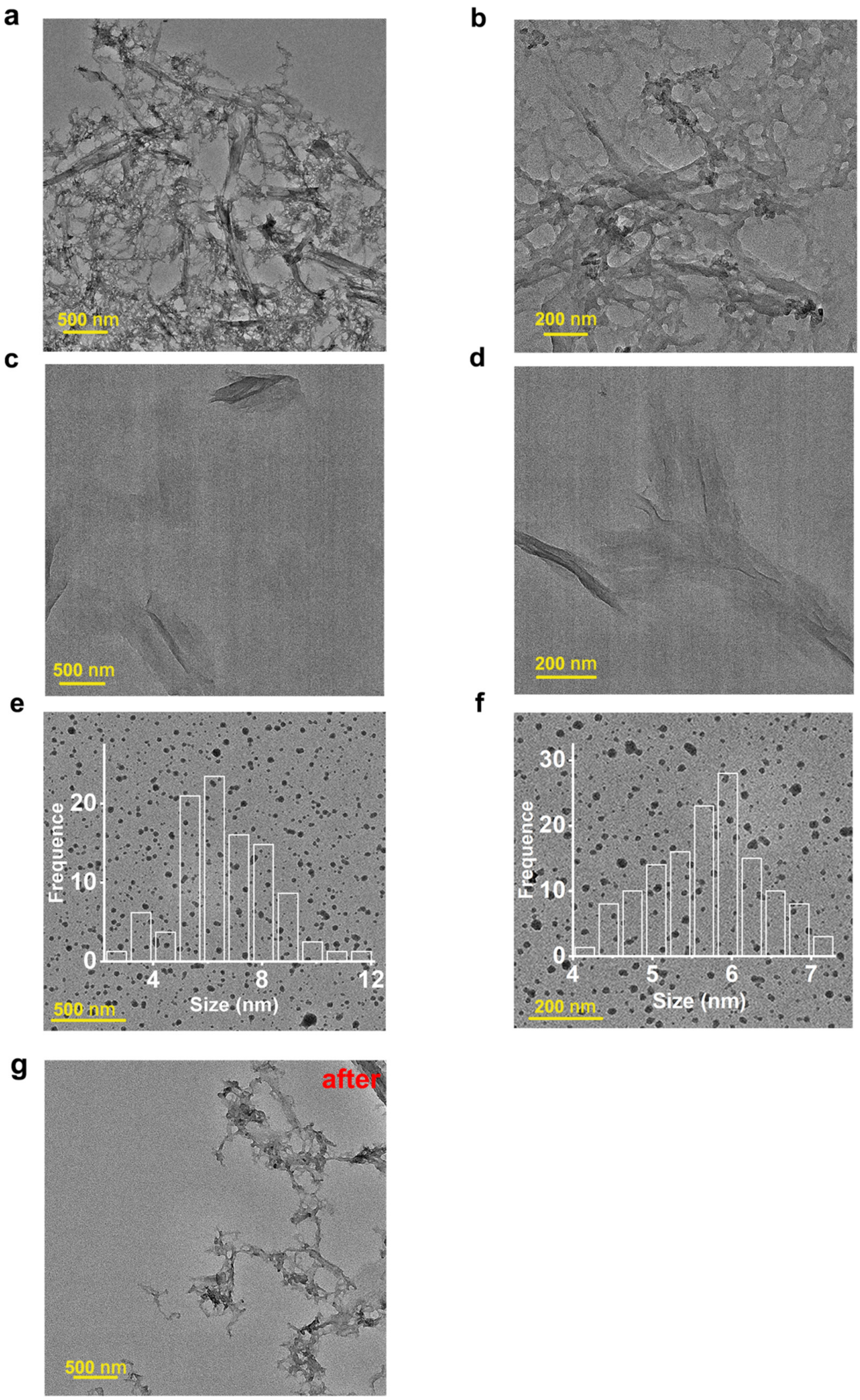

Figure S3 TEM images of QCDs (a,b), Quer (c,d) and CDs (e,f) with different scales. (g) The comparative morphology of QCDs after the reaction.

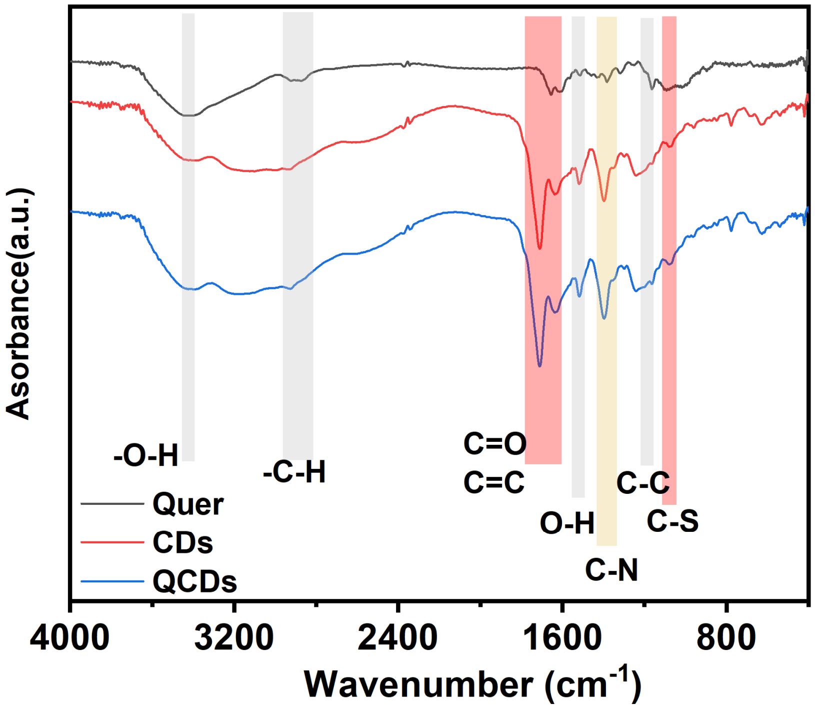

Figure S4 FTIR spectra of QCDs ,CDs and Quer.

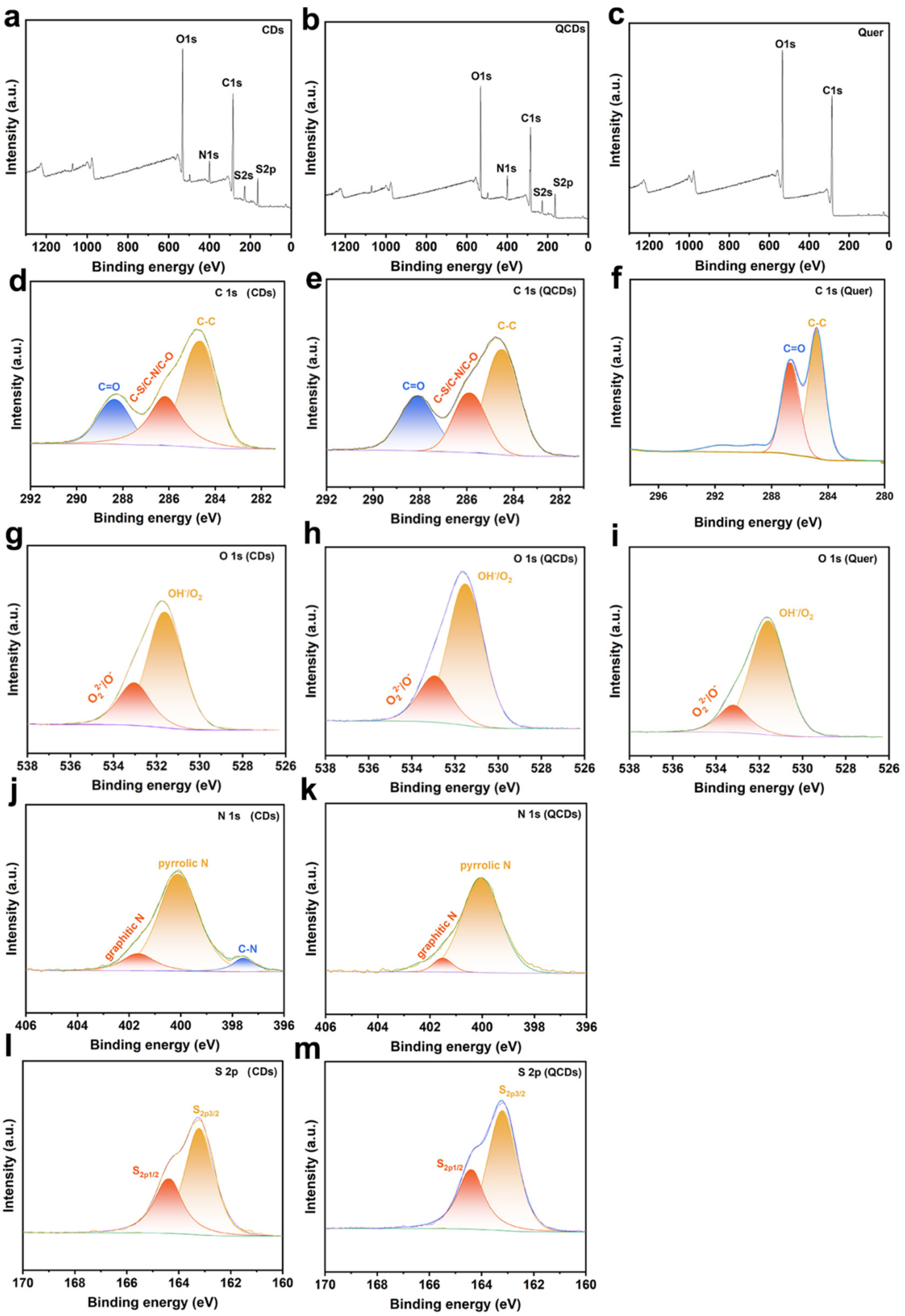

Figure S5 Full survey XPS spectra of CDs (a), QCDs (b) and Quer (c). High-resolution C 1s XPS spectra of CDs (d), QCDs (e) and Quer (f). High-resolution O 1s XPS spectra of CDs (g), QCDs (h) and Quer (i). High-resolution N 1s XPS spectra of CDs (j) and QCDs (k). High-resolution S 2p XPS spectra of CDs (l) and QCDs (m).

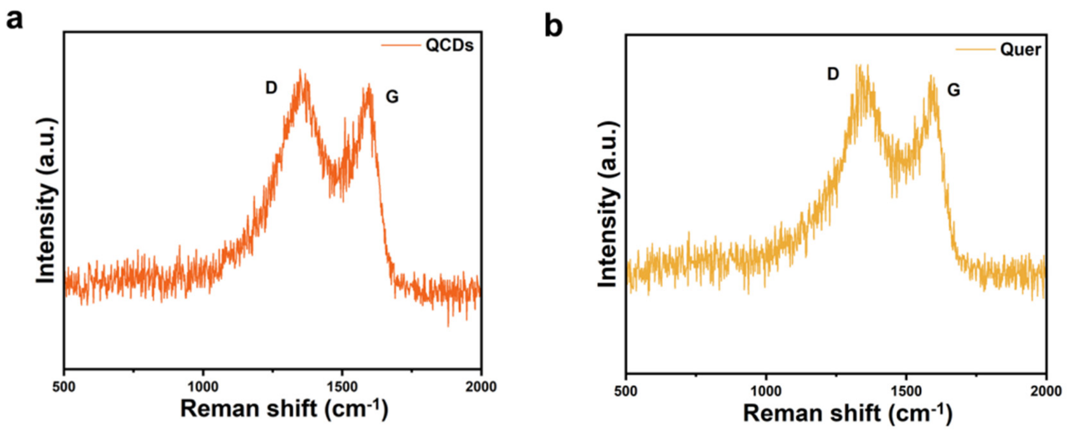

Figure S6 (a) Raman spectrum of QCDs, (b) Raman spectrum of Quer.

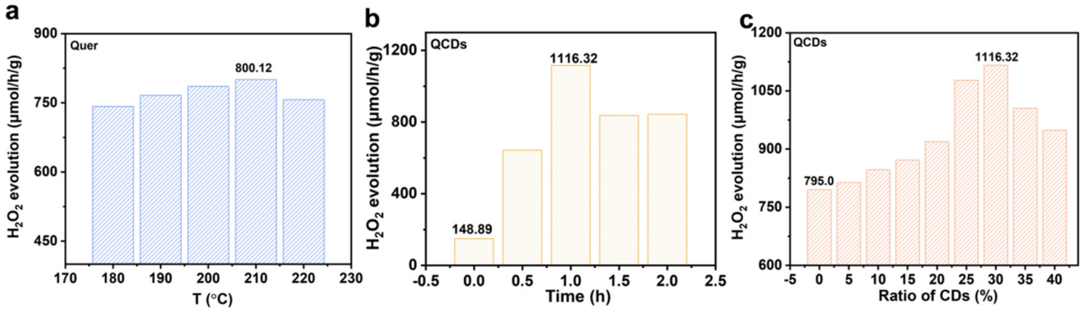

Figure S7 Photocatalytic generation of H_2O_2 using photocatalysts under different conditions. (a) Hydrothermal temperature, (b) reaction time, and (c) CDs dosages.

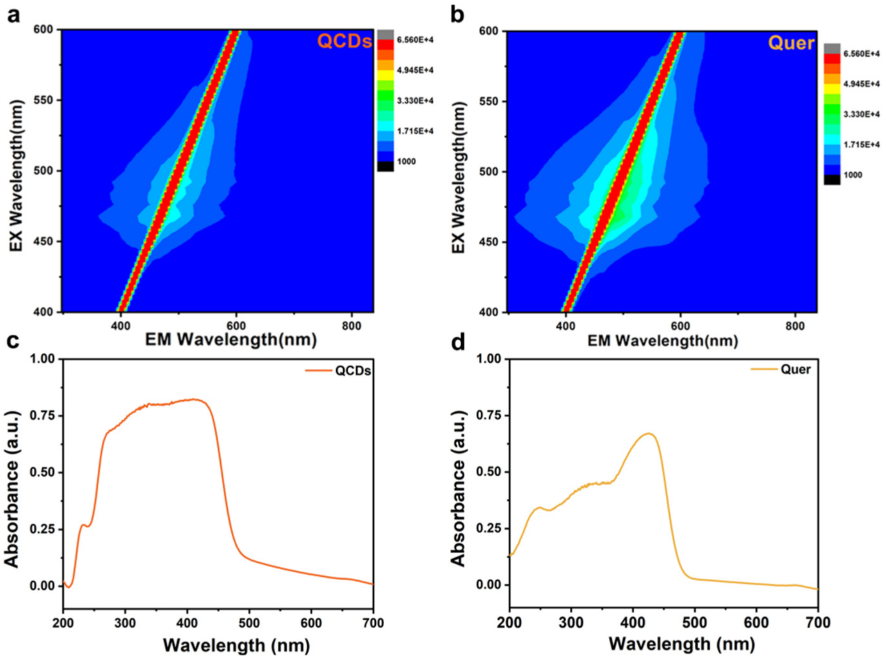

Figure S8 (a, b) PL emission spectra of QCDs and Quer. (c,d) UV-Vis absorption spectra of QCDs and Quer.

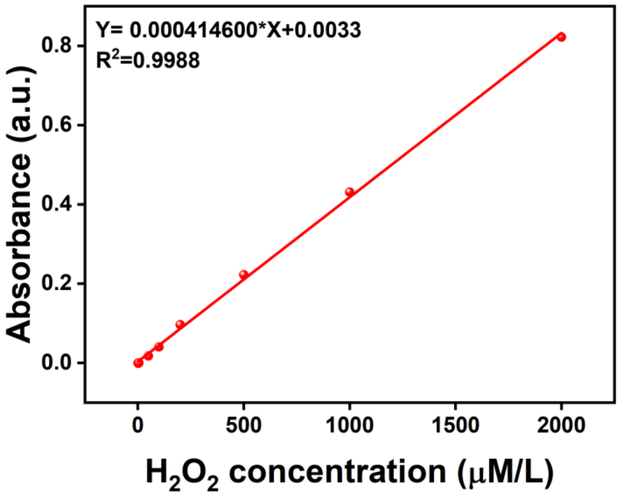

Figure S9 The fitted curve of UV-Vis absorbance at 412 nm *vs.* different H_2O_2 concentrations by the titanium sulfate method.

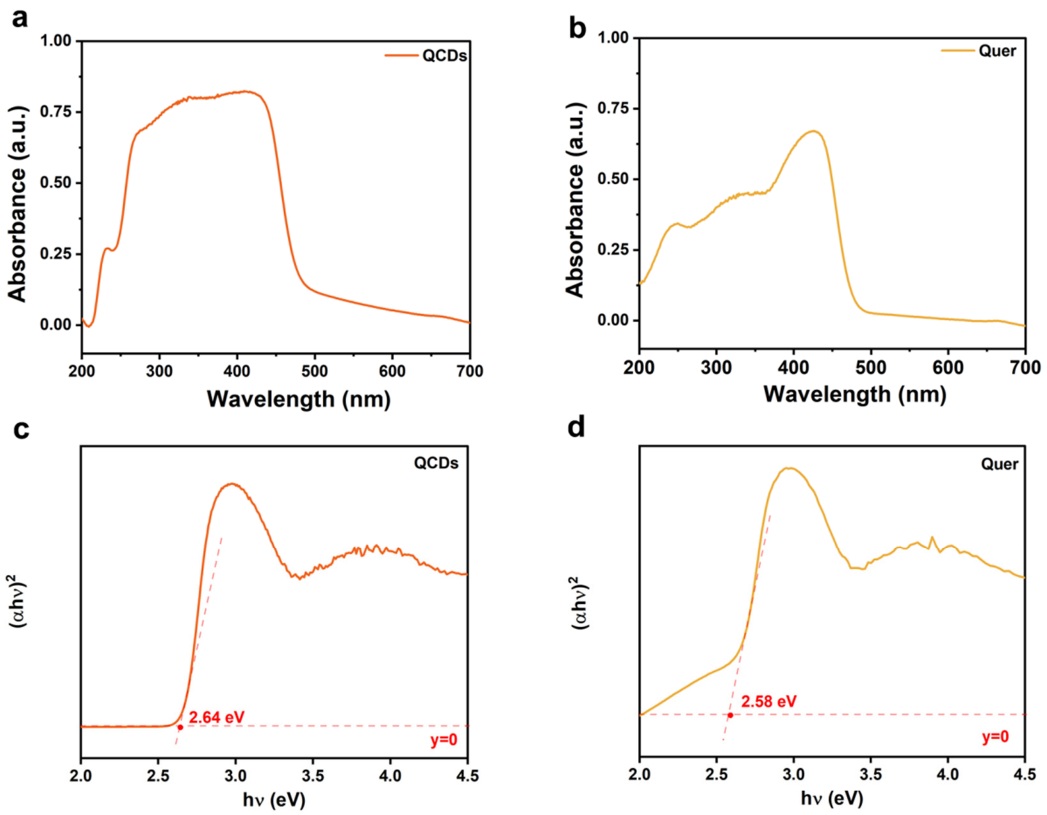

Figure S10 (a and b) UV-Vis absorption spectra of QCDs and Quer. Tauc plots for (c) QCDs and (d) Quer.

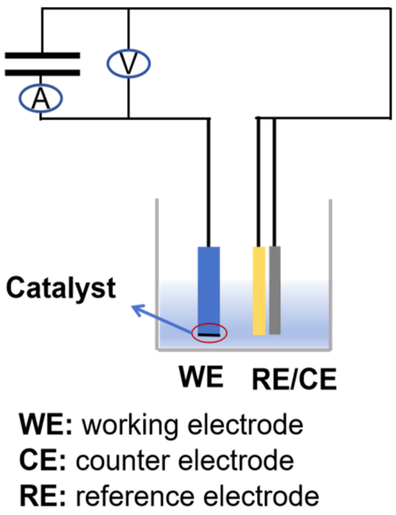

Figure S11 Schematic diagram of TPS.

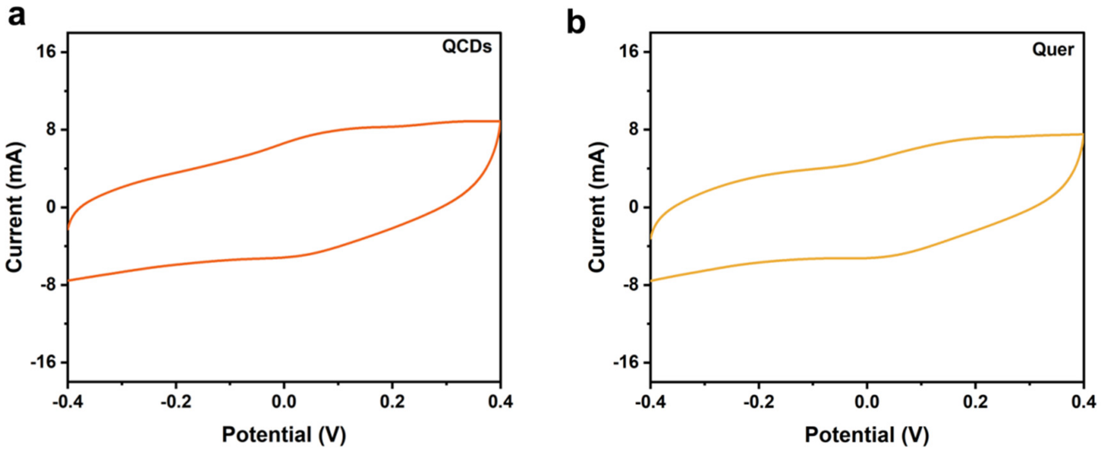

Figure S12 The CV curves of QCDs (a) and Quer (b) in 0.1 M Na_2SO_4 solution.

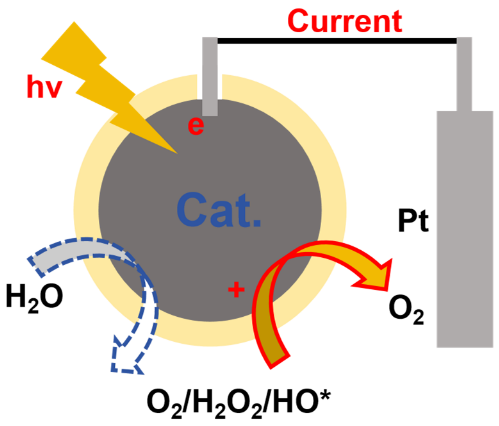

Figure S13 Schematic diagram of TPC.

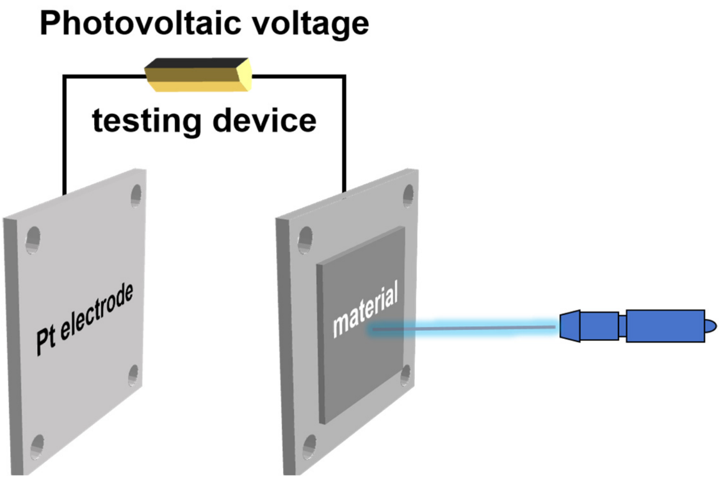

Figure S14 Schematic diagram of TPV.

Table S1. Performance comparison of carbon based photocatalysts.

Photocatalyst	Reaction solution	Photocatalytic Performance	Ref.
C-N-g-C_3N_4	H_2O	0.98 μmol·h^{-1}	[S1]
IO CN-Cv	EtOH	325.74 μmol·h^{-1}·g^{-1}	[S2]
CN-PDI	EtOH	573 μmol·h^{-1}·g^{-1}	[S3]
g-C_3N_4-BDI_{50}	EtOH	5.7 μmol·h^{-1}·g^{-1}	[S4]
OE-CQDs	EtOH	356.86 μmol·h^{-1}·g^{-1}	[S5]
**QCDs**	**H_2O**	**1116.32 μmol·h^{-1}·g^{-1}**	**This work**

References

S1. Fu, Y.; Liu, C.; Zhang, M.; Zhu, C.; Li, H.; Wang, H.; Song, Y.; Huang, H.; Liu, Y.; Kang, Z. Photocatalytic H_2O_2 and H_2 generation from living chlorella vulgaris and carbon micro particle comodified g-C_3N_4. *Adv. Energy Mater.* **2018**, *8*, 1802525.

S2. Lei, J.; Chen, B.; Lv, W.; Zhou, L.; Wang, L.; Liu, Y.; Zhang, J. Robust photocatalytic H_2O_2 Production over Inverse Opal g-C_3N_4 with carbon vacancy under visible light. *ACS Sustainable Chem. Eng.* **2019**, *7*, 16467-16473.

S3. Shiraishi, Y.; Kanazawa, S.; Kofuji, Y.; Sakamoto, H.; Ichikawa, S.; Tanaka, S.; Hirai, T. Sunlight-driven hydrogen peroxide production from water and molecular oxygen by metal-free photocatalysts. *Angew. Chem. Int. Ed.* **2014**, *53*, 13454-13459.

S4. Kofuji, Y.; Ohkita, S.; Shiraishi, Y.; Sakamoto, H.; Tanaka, S.; Ichikawa, S.; Hirai, T. Graphitic carbon nitride doped with biphenyl diimide: Efficient photocatalyst for hydrogen peroxide production from water and molecular oxygen by sunlight. *ACS Catal.* **2016**, *6*, 7021-7029.

S5. Do-Yeon Lee; Z. Haider; S. K. Krishnan; T. Kanagaraj; S. H. Son; J. Jae; J. R. Kim; P. S. M. Kumar; H. Kim. Oxygen-enriched carbon quantum dots from coffee waste: Extremely active organic photocatalyst for sustainable solar-to-H_2O_2 conversion. *Chemosphere* **2024**, *361*, 142330.
